# On the Equivalence of Gibbs, Boltzmann, and Thermodynamic Entropies in Equilibrium and Nonequilibrium Scenarios

**DOI:** 10.3390/e27101055

**Published:** 2025-10-10

**Authors:** Anil A. Bhalekar, Vijay M. Tangde

**Affiliations:** Department of Chemistry, Rashtrasant Tukadoji Maharaj Nagpur University, Nagpur 440 033, India

**Keywords:** Boltzmann entropy, Gibbs entropy, entropy, CSTR, statistical mechanics, entropy extremization, nonequilibrium thermodynamics

## Abstract

In this presentation, we have identified the domain of equivalence amongst the Boltzmann, Gibbs, and thermodynamic entropies. In this domain, ergodicity is followed even for (i) all nonequilibrium steady states and (ii) those time-dependent nonequilibrium states belonging to it. The condition of this domain is either that the rate of entropy change is zero or its magnitude is exceedingly small. Its implication is that, in this domain, Jaynes’ principle of maximum entropy estimate also holds. Outside this domain, the said equivalence among three entropies is not feasible, and the operation of the Jaynes’ principle of maximum entropy estimate does not remain of practical utility.

## 1. Introduction

Boltzmann and Gibbs entropies play a central role in forming a bridge between microscopic and macroscopic properties of matter. The well-established branch named statistical thermodynamics for equilibrium states is a testimony of the soundness of this bridge [1,2,3,4]. However, there is a basic difference between the ways these two entropies are computed. In computing the Boltzmann entropy, the number of microstates of a given system is used, and for this purpose, the systems with non-interacting molecules are appropriate. Meanwhile, for computing the Gibbs entropy, the microstates of an ensemble are employed. An ensemble is constructed with a large number of replicas of the system but are in different quantum states commensurate with the thermodynamic conditions operating on it. Thus, the restriction of non-interacting molecules of the former gets removed in the latter. In 1965, Jaynes [5] elucidated this fact and clearly brought forward the distinction between these two entropies with a proof that the difference between the two vanishes when the system is composed of non-interacting molecules. Otherwise, the computation of Boltzmann entropy gives a lower value than that obtained using Gibbs entropy, and the equilibrium thermodynamic entropy matches well with the Gibbs entropy. Thus, this bridging is based on the equivalence of Gibbs and thermodynamic entropies in the equilibrium scenario, whereas it is obvious that, in the case of systems with non-interacting molecules, no distinction exists among these three entropies. We also recall that in the above stated equivalence, one uses the maximized values of Boltzmann and Gibbs entropies. The said maximization is carried out in the statistical mechanical premises and uses the mathematics of chance. That is, this method of maximization has nothing to do with the second law of thermodynamics. De Groot and Mazur [6] have also analyzed the equivalence between these entropies based on the fluctuations about equilibrium states, wherein they use the standard method of maximization of entropy, and their results are in tune with Jaynes’ above-stated demonstration for equilibrium states. However, in thermodynamics, we do have maximization of entropy at the final equilibrium state under the adiabatic condition, but this maximization is a macroscopic observation valid only for the end state of corresponding irreversible trajectory. Hence, its premises of operation, the phenomenological, is distinctly different than the above-stated statistical mechanical premises. We also recall that, in 1957, Jaynes propounded the rule of maximum entropy estimates for information-theoretic entropy [7,8]. However, this rule has been in use, since even before it was established in 1957, while computing Boltzmann and Gibbs entropies of an equilibrium state by resorting to the mathematics of chance.

However, with the advent of nonequilibrium thermodynamics [6,9,10,11,12], it becomes natural to inquire whether a similar equivalence does exist in the nonequilibrium scenario? Also, what will be the status of Jaynes’ maximum Boltzmann and Gibbs entropy estimates when a system is in nonequilibrium?

For answering the above questions, we have adopted an approach that involves the use of a continuously stirred tank reactor (CSTR) with *n*-number of independent chemical reactions taking place within it. Then, we proceed to construct isentropic hypersurfaces for the nonequilibrium steady states and isentropic planes for equilibrium states appropriate for the considered CSTR. Based on these hypersurfaces and planes, we describe various reversible paths and irreversible trajectories, and then a few relevant thermodynamic characteristic relations are recalled in terms of the variation of thermodynamic, Boltzmann, and Gibbs entropies. With this background, the question of equivalence of three entropies in the nonequilibrium scenario is also discussed. A scope of the validity of the Jaynes’ maximum entropy estimates for Boltzmann and Gibbs entropies in the nonequilibrium domain is analyzed. Finally, we present concluding remarks.

This paper has been written on an invitation to contribute in the Special Issue of *entropy* on the occasion of 50 years of the finite time thermodynamics [13,14,15,16]. This paper deals with a very basic statistical thermodynamic aspect, the ergodicity, extending to nonequilibrium situations. Hence, this subject is of relevance for all nonequilibrium thermodynamic formulisms, including the finite time thermodynamics.

## 2. Continuously Stirred Tank Reactor with Several Independent Chemical Reactions

**Figure 1:**
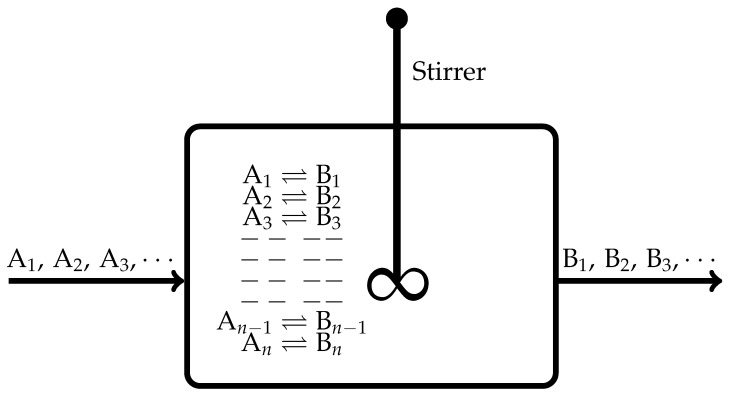
A schematic depiction of a CSTR in which *n*-number of independent chemical reactions are proceeding at non-vanishing rates. The flow-in of the reactants $A_{1},\;A_{2},\;A_{3},\;\ldots$ and the flow-out of the products $B_{1},\;B_{2},\;B_{3},\;\ldots$ can be controlled as per the state of the system that we wish to study. For example, approach towards an equilibrium state or a nonequilibrium steady state or when the system has attained the final equilibrium or nonequilibrium steady state.

We find that the continuously stirred tank reactor (CSTR) shown in Figure 1 is a simple system, with an advantage that it can be operated in different thermodynamic conditions, such as maintained at an equilibrium or a nonequilibrium steady state, which allows it to evolve irreversibly either to an equilibrium or a nonequilibrium states, etc. It is a spatially uniform and homogeneous system.

In the following discussion, the standard thermodynamic relations suitable for the CSTR of Figure 1 are being used without spelling out their background in details (refer to any standard thermodynamic textbook, for example [17,18,19,20]). For example, we recall the following ones:

- When all the flows are stopped, the CSTR depicted in Figure 1 evolves towards an equilibrium state. During this evolution, according to the De Donderian Equation [20], we have the following functional dependence:
  (1) $$S\left(t\right)=S\left(U\left(t\right),\;V\left(t\right),\;\{\xi_{r}\left(t\right)\}\right)$$
  where *S* is the entropy, *U* is the internal energy, *V* is the volume of the reactor, $\left\{\xi_{r}\right\}$ are the respective extents of advancement of the chemical reaction identified with the running subscript *r*, and *t* is time.
- When this CSTR attains an equilibrium state, the functional dependence of Equation (1) transforms to the following:
  (2) $$S=S\left(U,\;V\right)=constant,$$
  That is, now we have $\xi_{r}=\xi_{r}\left(U,\;V\right)$ for all $\left\{\xi_{r}\right\}$, implying they do not remain independent thermodynamic variables in the case of chemically reacting systems.
- On attainment of a nonequilibrium steady state, the functional dependence of Equation (1) becomes time-independent:
  (3) $$S=S\left(U,\;V,\;\{\xi_{r}\}\right)=constant.$$

## 3. Geometrical Aspects of Thermodynamic Entropy Based on Isentropic Hypersurfaces Housing Nonequilibrium Steady States

Since the CSTR shown in Figure 1 constitutes a spatially uniform and homogeneous system, when evolving towards an equilibrium state or a nonequilibrium state, the following entropy balance is obeyed [6,9,10]:

(4) $$\frac{dS}{dt}=\frac{d_{e}S}{dt}+\frac{d_{i}S}{dt}$$

where $\frac{d_{e}S}{dt}≷0$ is the rate of exchange of entropy, and $\frac{d_{i}S}{dt}\geq 0$ is the positive definite rate of entropy production. The attainment of a nonequilibrium steady state is described by the following:

(5) $$\frac{dS}{dt}=0\;⟹\;\frac{d_{i}S}{dt}=-\frac{d_{e}S}{dt}\geq 0$$

That is, at a nonequilibrium steady state, the rate of entropy production equals the rate of entropy driven out of the system. The operative functional dependence for the considered CSTR reads as that given in Equation (3).

**Figure 2:**
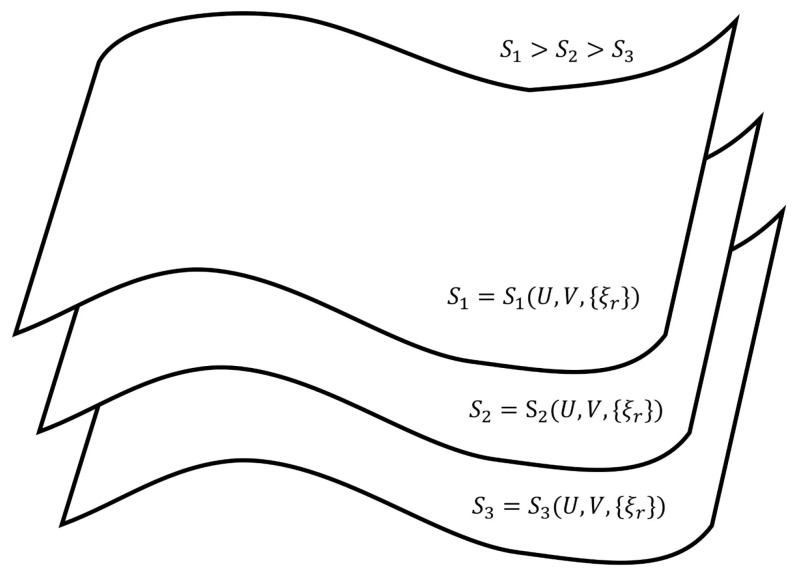
A schematic representation of isentropic hypersurfaces in the $\left(U,\;V\;\left\{\xi_{r}\right\}\right)$ space with entropy as the vertical axis that is with increasing entropy ($S_{1}>S_{2}>S_{3}$). All paths lying exclusively on a given hypersurface are reversible isentropic ones composed of a succession of nonequilibrium steady states, but they are not reversible adiabatic ones because the rate of entropy exchange is non-zero. A reversible non-isentropic path between two end nonequilibrium steady states will be across the isentropic hypersurfaces composed of a succession of nonequilibbrium steady states of varying magnitudes of entropy, which also can be traversed in both the directions. Irreversible non-isentropic paths (trajectories) will also be across the isentropic hypersurfaces but will be accompanied by the variation of entropy as well as both entropy production and entropy exchange.

Geometrically, the expression in Equation (3) produces an isentropic hypersurface in the thermodynamic space determined by the coordinates $\left(U,\;V,\left\{\xi_{r}\right\}\right)$. This hypersurface houses time-independent nonequilibrium steady states of identically same values of entropy. Next, by varying the value of the constant in Equation (3), we obtain a family of non-intersecting isentropic hypersurfaces of different values of entropy and those we can stack one over the above in the increasing value of entropy (One of the present authors had earlier identified isentropic hypersurfaces for nonequilibrium steady states by proposing an universal inaccessibility principle. For its details the reader may refer to [21,22,23,24]). This we have depicted in Figure 2.

**Figure 3:**
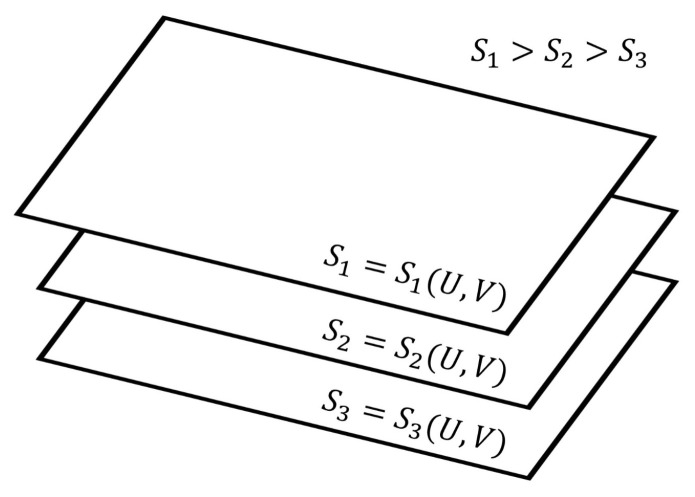
A schematic representation of isentropic planes in the (U,V) space with entropy as the vertical axis that is with increasing entropy ($S_{1}>S_{2}>S_{3}$). All curves lying exclusively on a given plane are reversible isentropic ones, which are reversible adiabatic planes too. A non-isentropic (that is non-adiabatic) reversible path will be across the isentropic planes and can be traversed in both the directions, whereas irreversible adiabatic paths will also be across the isentropic planes but will be in the direction of increasing entropy because the change in entropy remains equal to the amount of entropy production. However, the natural direction of non-adiabatic irreversible path (necessarily will be across the isentropic planes) cannot be prescribed in terms of increasing entropy because the sign of dS on such paths also has a contribution from the exchange of entropy with its surrounding but with no definite sign.

The transformation of the functional dependence of Equation (3) to that of Equation (2) transforms the non-intersecting isentropic hypersurfaces shown in Figure 2 to the non-intersecting isentropic planes depicted in Figure 3. These isentropic planes house equilibrium states (for a detailed account on the isentropic hypersurfaces for equilibrium states, the reader is directed to refer [18,25,26]).

**Figure 4:**
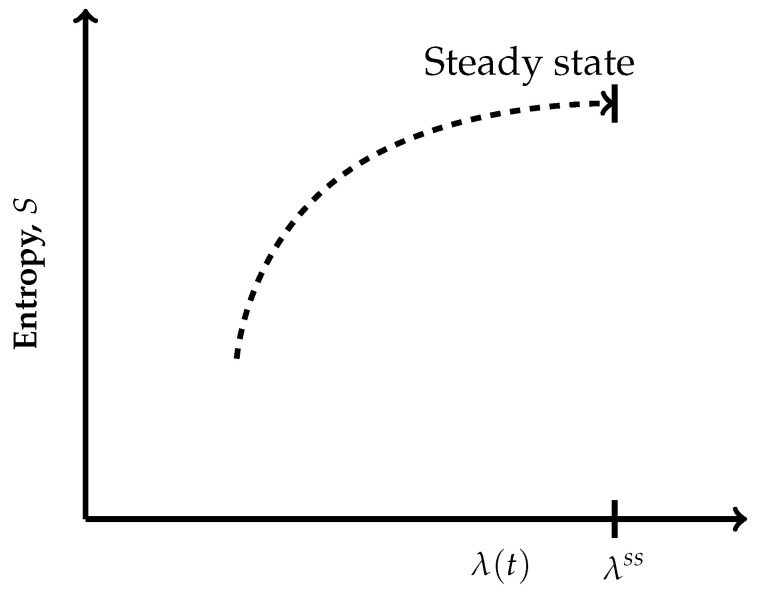
Schematic depiction of the variation of entropy in a CSTR at constant *U* and *V* with non-zero rate of entropy driven out, which is dominated by the rate of entropy production near nonequilibrium steady state. Through the parameter λ, we quantify the imbalance between the rate of entropy production and the rate of entropy driven out, whose value is $\lambda^{ss}$ at the nonequilibrium steady state. A simple expression in the vicinity of the nonequilibrium steady state is $S=S^{ss}\exp\left(k(\lambda^{ss}-\lambda )\right)$, whereas when the final state is in equilibrium because the rate of entropy exchange remains zero, we have to use the extent of advancement of reaction ξ as the parameter instead of λ. Therefore, in the vicinity of the equilibrium state, we have the expression $S=S^{e}\exp\left(k(\xi^{e}-\xi )\right)$ (hence, during the course of approaching an equilibrium state, the horizontal axis should be interpreted in terms of ξ(t) and $\xi^{e}$). In these two expressions, *k* is the kinetic constant. This trajectory obviously is of increasing entropy, and the final state is a nonequilibrium steady state. However, if we impose the condition of a zero rate of entropy exchange, then the CSTR behaves as if evolving under isolation/adiabatic condition; hence, the final state will be an equilibrium state of highest entropy. Therefore, the same curve represents this situation too. The trajectory has been depicted as a dashed curve to emphasize that it passes through a succession of time-dependent nonequilibrium states.

**Figure 5:**
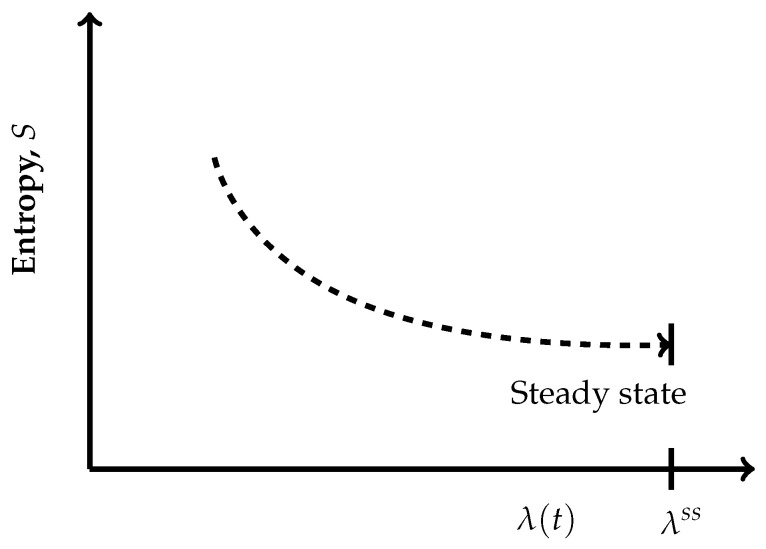
Schematic depiction of the variation in entropy in a CSTR at constant *U* and *V* with a non-zero rate of entropy driven out that dominates the rate of entropy production near the nonequilibrium steady state. Through the parameter λ, we quantify the imbalance between the rate of entropy production and the rate of entropy driven out, whose value is $\lambda^{ss}$ at the nonequilibrium steady state. A simple expression in the vicinity of the nonequilibrium steady state is $S=S^{ss}\exp\left(-k\left(\lambda^{ss}-\lambda \right)\right)$, where *k* is the kinetic constant. This trajectory obviously is of decreasing entropy, and the final state is a nonequilibrium steady state. The trajectory has been depicted as a dashed curve to emphasize that it passes through a succession of time-dependent nonequilibrium states.

Based on the isentropic hypersurfaces in Figure 2 and the isentropic planes in Figure 3, we arrive at the following thermodynamic description of various paths and trajectories:

1. The paths through a succession of nonequilibrium steady states of a given hypersurface of Figure 2 are the reversible isentropic paths. Their thermodynamic description in the De Donderian settings [20] reads as follows:
  (6) $$dS=0=\frac{1}{T}dU+\frac{p}{T}dV+\sum_{r}\frac{A_{r}}{T}d\xi_{r}$$
  where *p* is the pressure, *T* is the temperature and $\left\{A_{r}\right\}$ are the respective chemical affinities.
  The paths through a succession of nonequilibrium steady states across the hypersurfaces in Figure 2 are also reversible ones but are non-isentropic, as the entropy does not remain constant. Its description in the present case is as follows:
  (7) $$dS=\frac{1}{T}dU+\frac{p}{T}dV+\sum_{r}\frac{A_{r}}{T}d\xi_{r}\neq 0$$
  Recall that, by definition, a thermodynamically reversible path is a limiting case but never realized in practice; hence, they are termed as quasi-static paths, which, in principle, can be traversed in either direction. Also, since such paths are prescribed as the ones connecting infinitesimally close successions of time-independent nonequilibrium states, they cannot be termed irreversible paths, though the rate of entropy production remains non-zero. Earlier too, Keizer coined such reversible paths through a succession of nonequilibrium steady states [27,28,29] in his version of statistical thermodynamics of nonequilibrium processes based on the fluctuations of nonequilibrium steady states. On such paths, the complete compensation of the rate of entropy driven out and the rate of entropy production, as stated in Equation (5), is maintained (In biological systems an example approaching to a reversible path through a succession of nonequilibrium steady states is often presented. For example, biochemical reactions in biological cells, which are usually modelled as undergoing in a CSTR, operate under the nonequilibrium steady state conditions. And often other physico-chemical processes of a biological system are cooperatively associated with them. It has been observed that in biological systems it is not always possible to maintain the existence of the same nonequilibrium steady state continuously, but the system reversibly shifts to a new nonequilibrium steady state which gradually returns to the original one. This transition is considered as very close to a reversible passage through nonequilibrium steady states. That is why, the other biological processes dependent on this nonequilibrium steady state remain practically unhampered).
  On the other hand, the isentropic planes depicted in Figure 3 consist of equilibrium states. Therefore, when the thermodynamic reversible paths, through a succession of equilibrium states, are prescribed, then on such paths, no entropy production exists. Hence, in this case, the non-isentropic paths are described by $dS=d_{e}S≷0$ and Equation (7), whereas the isentropic paths follow $dS=d_{e}S=0$, with the following explicit description:
  (8) $$dS=0=\frac{1}{T}dU+\frac{p}{T}dV.$$
2. The thermodynamic irreversible trajectories are the ones in which the system leaves an isentropic hypersurface (an isentropic plane), attains a nearby time-dependent nonequilibrium state, and thereafter marches through a succession of time-dependent nonequilibrium states. They can be of two types: (1) in this category, the end state is a time-independent state (a nonequilibrium steady state or an equilibrium state), and (2) in this category, we club all those trajectories not converging to a steady state. In the first category, ultimately, the system attains the state of $\frac{dS}{dt}=0$. The variation of entropy during the attainment of this final state at constant *U* and *V* has two options. Recall that, by definition on an isentropic hypersurface, there is an exact compensation of entropy production and entropy driven out, as described in Equation (5). Therefore, for initiation of an irreversible process, the system has to leave an isentropic hypersurface. This happens only when the balance between the rate of entropy production and the rate of entropy driven out breaks down. Therefore, if the rate of entropy driven out dominates over the rate of entropy production, then the system follows the trajectory of decreasing entropy, whereas when the rate of entropy production dominates over the rate of entropy driven out, then during corresponding evolution of the system, its entropy increases. At this stage of our discussion, we introduce a parameter λ, through which we quantify the said imbalance between the rate of entropy production and the rate of entropy driven out. This parameter is different than the extent of the advancement of reaction ξ. This is so because the said imbalance also contains contributions to the rate of entropy production and the rate of entropy driven out by the non-steady state fluxes in and out of reactants and product molecules. When entropy is decreasing, the operative and simple expression very close to the nonequilibrium steady state is $S=S^{ss}\exp\left(-k\left(\lambda^{ss}-\lambda \right)\right)$, whereas in the case of increasing entropy, we have the simple expressions $S=S^{ss}\exp\left(k(\lambda^{ss}-\lambda )\right)$. However, when the system is attaining an equilibrium state, implying that the rate of entropy exchange remains zero, we have the expression $S=S^{e}\exp\left(k(\xi^{e}-\xi )\right)$. In the above three expressions, *k* is the kinetic constant, and in first two expressions, the superscript ss denotes a nonequilibrium steady state. Moreover, it is easy to comprehend that as the nonequilibrium steady state is approached, the existence time of the time-dependent nonequilibrium states goes on increasing and becomes sufficiently long in the close vicinity of it. The corresponding trajectories near a steady state appear as depicted in Figure 4 and Figure 5. When Figure 4 is used for the evolution to an equilibrium state, the parameters ξ(t) and $\xi^{e}$ need to be used instead of λ(t) and $\lambda^{ss}$.
  Thus, we observe from Figure 4 and Figure 5 that at $\lambda^{ss}$ we have
  (9) $${\left(\frac{\partial S}{\partial \lambda }\right)}_{U,\;V,\;\lambda^{ss}}=0,$$
  Which is the condition of extremization of entropy at the nonequilibrium steady state. When the final state is an equilibrium state, the variation of entropy will be as depicted in Figure 4, but then it requires the use of ξ(t) and $\xi^{e}$ instead of λ(t) and $\lambda^{ss}$ in this figure, re-expressing Equation (9) as ${\left(\partial S/\partial \xi \right)}_{U,\;V,\;\xi^{e}}=0$. However, away from the position $\lambda^{ss}$ on these curves, say at $\lambda^{\prime }$, we have
  (10) $${\left(\frac{\partial S}{\partial \lambda }\right)}_{U,\;V,\;\lambda^{\prime }}\neq 0,$$
  And away from an equilibrium state at a position $\xi^{\prime }$ on the curve of Figure 4, this condition reads as ${\left(\partial S/\partial \xi \right)}_{U,\;V,\;\xi^{\prime }}\neq 0$. It illustrates that there is no possibility at all to have ${\left(\partial S/\partial \xi \right)}_{U,\;V,\;\lambda^{\prime }}=0$ and ${\left(\partial S/\partial \xi \right)}_{U,\;V,\;\xi^{\prime }}=0$ for a time-dependent nonequilibrium state even for the situations which are in very close proximity to a steady state. Hence, at the phenomenological level, we can neither claim them as being of maximum entropy nor of minimum entropy with regard to their neighboring states on the trajectory.

Notice that the partial derivatives of the thermodynamic entropy appearing in Equations (9) and (10) belong to the phenomenological premises. We will see in Section 4 that the same equations hold true for Boltzmann and Gibbs entropies in the phenomenological premises.

## 4. Statistical Mechanical Versus Phenomenological Aspects and the Question of Equivalence of Entropies

We recall the following standard expressions: (1) the Boltzmann entropy, *S*:

(11) $$S=k_{B}\ln W$$

where $k_{B}$ is the Boltzmann constant, and *W* is the thermodynamic probability that measures the number of microstates commensurate with the given macrostate of the system; and (2) the Gibbs entropy, *S*, which is based on the ensemble averaging, which reads as follows:

(12) $$S=k_{B}\ln\Omega$$

where Ω is the ensemble based number of microstates. Conventionally, in the case of equilibrium states, the mathematics of chance is employed, which establishes $W\approx W_{max}$ and $\Omega \approx \Omega_{max}$; hence, correspondingly, the Boltzmann and Gibbs entropies calculated using Equations (11) and (12) are the maximized values. It means that while computing Boltzmann and Gibbs entropies, all the microstates are counted. In the case of the ensemble method, it is termed as an ensemble averaged value. However, to grant it a physical status, the concept of the time averaging is used, which means that in sufficiently large time intervals, the system detours all the members of $\Omega_{max}$. The experimentally measured quantities are the results of this detouring. Hence, the time-averaged and ensemble-averaged values are equated, which in statistical mechanics is described as ergodicity [2,3]. With this background, we further assume that the maximum entropy estimates, proposed by Jaynes [7,8] for information-theoretic entropy, can be extended for computing the Boltzmann and Gibbs entropies of a system when it is in (i) a nonequilibrium steady state and (ii) a time-dependent nonequilibrium state, which is very close to a time-independent state (We have not used different symbols for Boltzmann, Gibbs and thermodynamic entropies in the present discussion except for the alternative Gibbs entropy (cf. Equation (14))).

Jaynes has already demonstrated the equivalence of Gibbs and thermodynamic entropies [7] for all systems in equilibrium. The reason is that both take into account all contributions to internal energy, including the existing non-ideality. When a system is in equilibrium and contains monatomic ideal gases, we have equivalence amongst Boltzmann, Gibbs, and thermodynamic entropies. Also, notice that the maximization used in Equations (11) and (12) is with regard to the neighboring *W*s and Ωs of lower magnitude. These lower value Ωs (*W*s) belong to the same set of say $\left(U,\;V,\;\left\{\xi_{r}\right\}\right)$ and hence cannot be thought as the neighboring points on the considered irreversible trajectory. The use of maximized Ω is physically permitted when an equilibrium state is of the time-independent type described, say by $\frac{dS}{dt}=0$, and therefore the system has ample time at its disposal to detour all of them. This condition shown in Figure 4 is followed at the maximum irrespective of whether the system is ideal or not.

In the sequel, we recall that the nonequilibrium steady states are also time-independent states described by $\frac{dS}{dt}=0$ and correspond to the extremum of Figure 4 and Figure 5. In this case too, the system has at its disposal ample time to detour all the microstates counted by $\Omega_{max}$ in non-ideal systems and by $W_{max}=\Omega_{max}$ in an ideal system. This means that the ergodicity is obeyed in this case too. Hence, in the case of nonequilibrium steady states too, we have equivalence amongst these three entropies when the system consists of ideal gases and between Gibbs and thermodynamic entropies when the system consists of non-ideal components. Thus, we see that Equation (9) also holds for Gibbs and Boltzmann entropies when the system is at a nonequilibrium steady state.

Next we consider the time-dependent nonequilibrium states. For example, refer to the states lying on the trajectories away from the steady points, $\lambda^{e}$ ($\xi^{e}$), as shown in Figure 4 and Figure 5. They all, that is the points $\lambda^{\prime }$ ($\xi^{\prime }$), follow the expression of Equation (10) for thermodynamic entropy because they are not steady states described by $\frac{dS}{dt}\neq 0$. This raises the question of whether an equivalence amongst these three entropies can be assumed. The answer lies in ascertaining whether the system can detour all the microstates of $\Omega_{max}$ and, if applicable, those counted by $W_{max}$ before the next time-independent state is attained. In principle, the answer will be no, but as we get closer to the final time-independent state, we see a decrease in the magnitude of $\frac{dS}{dt}$, and it will be exceedingly small in the close vicinity of the former. In such cases, the system will have sufficient time at its disposal to detour almost all microstates counted by $\Omega_{max}$ ($W_{max}$); that is, the ergodicity remains valid. Thus, for all such time-dependent nonequilibrium states, the said equivalence will come within the physically acceptable domain, of course within the limits of experimental errors or accuracy, even though the expression of Equation (9) is not followed. But a validity of the expression of Equation (10) for Boltzmann and Gibbs entropies does exist. In practice, such situations are numerous, and all of them are not very close to a steady state. However, calculations need to be carried out of transport properties using Gibbs ensemble method to produce suitable examples. However, an encouraging fact is that, in the kinetic theory of gases, these transport properties in a dynamic system have been computed, and the results satisfactorily match with those measured experimentally [30,31,32,33].

On the other hand, the time-dependent nonequilibrium states with a significantly large magnitude of $\frac{dS}{dt}$ have short existence times. Since their existence time does not allow the system to detour all the members of the computed $\Omega_{max}$, implying no feasibility of obeying ergodicity, the calculated Gibbs entropy from the physicality point of view cannot be assigned to such short-lived states even though their instantaneous macroscopic conditions are used in the computation. This perspective has been previously emphasized by one of the present authors [34]. Hence, in such situations, not only the said equivalence of entropies but also the validity of Jaynes maximum entropy estimates are of doubtful practical utility.

Of course, there are countless examples of systems in short-lived states. It means that such systems, when in short-lived states, do detour a certain number of microstates, which will be less than the value of $\Omega_{max}$ calculated using mathematics of chance for the same thermodynamic conditions. The thermodynamic conditions for a given nonequilibrium state are based on the independent thermodynamic variables appropriate for it. In extended irreversible thermodynamics [12,23,35,36,37,38,39,40,41,42,43,44], the physical fluxes are raised to the status of thermodynamic variables. Thus, the entropy dependence therein, for example, reads as follows:

(13) $$s=s\left(u,\;v,\;\left\{x_{k}\right\},\;q,\;\Pi ,\;\left\{J_{k}\right\}\right)$$

where *s* is the per unit mass local entropy, *u* is the per unit mass local internal energy, *v* is the local specific volume, $\left\{x_{k}\right\}$ are the local mass fractions of the components, q is the local heat flux, Π is the local dissipative momentum flux tensor, and $\left\{J_{k}\right\}$ are the local diffusion fluxes. All these quantities, in general, are position- and time-dependent. Therefore, the ensemble is constructed say for a particular instantaneous set $\left(u,\;v,\;\left\{x_{k}\right\},\;q,\;\Pi ,\;\left\{J_{k}\right\}\right)$, and then $\Omega_{max}$ is computed using the mathematics of chance. Now, suppose the ensemble is constructed for one of the very short-lived nonequilibrium states. In view of the transient nature of the state under consideration, the system will not have adequate time at its disposal to traverse all the members of $\Omega_{max}$—a case of non-compliance of ergodicity. Hence, the calculated maximized value of Gibbs entropy will be higher than the actual thermodynamic entropy. Therefore, instead of maximization, we need to devise a mathematical model to arrive at the appropriate and physically relevant value of Ω. Indeed, one may use some other nonequilibrium thermodynamic framework, say rational thermodynamics, finite time thermodynamics, Keizer’s version, thermodynamics with internal variables, or any other; the above assertion does not change because the basic problem being faced herein does not originate in the use of a thermodynamic framework but is due to the very short existence time of a nonequilibrium state; hence, it is the case of the nonfeasibility of traversing all the members of $\Omega_{max}$ by the system.

Therefore, in general, the maximized Gibbs entropy given by Equation (12) for short-lived nonequilibrium states will be higher than the actual thermodynamic entropy. This demonstrates why Jaynes’ maximum entropy estimate remains inapplicable to such short-lived nonequilibrium states.

Since our discussion involves the traditional definition of Gibbs entropy, as seen in Equation (12), it is pertinent also to consider an alternative Gibbs entropy, defined as follows:

(14) $$S_{G}\left(U\right)=k_{B}\ln\left(\sum_{U^{\prime }<U}\Omega \left(U^{\prime }\right)\right)$$

This definition can be used also for small systems consisting of tens of molecules. In a recent discussion [45], it is illustrated therein that in the case of equilibrium and for astronomically large values of Ω, $S_{G}$ coincides with the definition of Equation (12). However, as Ω decreases (corresponding to only tens of molecules), the non-equivalence between the two entropies surfaces out. In this limit, the physical meaning of say hot and cold crumbles down. However, in the case of time-independent states and the time-dependent nonequilibrium states, an additional condition for the said equivalence and nonequivalence will operate in terms of the magnitude of the time rate, $\frac{dS_{G}}{dt}$.

## 5. A Brief Note on Natural Fluctuations

The phenomena of fluctuations is common in all experimental measurements carried out on a system in a static condition or evolving with time. In equilibrium statistical thermodynamics, it is quantified in terms of standard deviation. When the Gibbs ensemble is used, the averaging is carried out over all the members of $\Omega_{max}$ (refer to any standard statistical mechanics and statistical thermodynamic textbooks, such as those cited in this paper).

Let us examine a system in equilibrium. In view of the above discussion, we envisage that the observed fluctuations will have two types of contributions if the system is chemically reactive. In the traditional description of fluctuations, they are the ones taking place at constant composition at equilibrium. However, when the system is chemically reactive, the extent of advancement of various chemical reactions will also fluctuate. However, this macroscopic fluctuation originates from the corresponding fluctuations in the population of quantum states of reacting partners. In other words, now the origin of fluctuations have contributions from two types of molecular collisions; one is non-chemically reactive type, and the other chemically reactive type. For its illustration, let us consider a canonical ensemble for chemically reactive components. In the present case too, the traditionally computed standard deviation, $\sigma_{U}$, in internal energy, *U*, will also be a complete description having the following expression [1]:

(15) $$\sigma_{U}=\sqrt{\bar{{\left(U-\bar{U}\right)}^{2}}}$$

Notice that the two averages involved in Equation (15) are computed by considering population in all the quantum states but the effective contribution to it is from the set of population effectively contributing to $\Omega_{max}$. However, a basic challenge is to devise an appropriate expressions of Ω for chemically reacting systems.

A similar argument also applies to the fluctuations about a nonequilibrium steady state because it is a time-independent state.

However, when we consider fluctuations of a time-dependent nonequilibrium state, similar arguments hold in the case of extremely small magnitude of $\frac{dS}{dt}$ because, in this case, ensemble averaging produces physically realistic results. Only in those cases where the magnitude of $\frac{dS}{dt}$ is significantly large and more, no physically meaningful statistical averaging is feasible, will the statistical computation of the standard deviation in fluctuations be of doubtful physical utility. This result is due to the non-compliance of ergodicity.

## 6. Concluding Remarks

In the present discussion, we have recalled the demonstration by Jaynes that (1), in general, there exists an equivalence between thermodynamic and Gibbs entropies, and (2) the equivalence between Boltzmann and Gibbs entropies exists only when the systems are composed of monatomic ideal gases. In Section 3, we have clearly illustrated by recalling the thermodynamic descriptions of extremization and maximization of thermodynamic entropy in terms of appropriate derivatives of entropy. For this purpose, we have used a specific and simple CSTR (described in Section 2) and generated corresponding isentropic hypersurfaces for nonequilibrium steady states and isentropic planes for equilibrium states. Thereafter, on combining phenomenological (of Section 3) and microscopic (of Section 4) descriptions, the following conclusions are reached:

1. In determining the equivalence between thermodynamic and statistical mechanically defined entropies, a crucial role is played by the ability of the system to detour all the members of the microstates of the system that is an ability to obey ergodicity.
2. The requirement stated above is meticulously met only when a system is in an equilibrium state but also in nonequilibrium steady state. This is so because they both are time-independent states. Thus, even though a nonequilibrium steady state belongs to the nonequilibrium regime, the equivalence between Gibbs and thermodynamic entropies is guaranteed. Of course, for the equivalence of Boltzmann entropy the absence of non-ideality is demanded.
3. In the case of time-dependent nonequilibrium states with an exceedingly small magnitude of $\frac{dS}{dt}$, the requirement stated in 1 above is practically met; hence, for them, the equilvalence between thermodynamic and Gibbs entropies exists.
4. For all time-dependent nonequilibrium states belonging to considerably large magnitudes of $\frac{dS}{dt}$, because the condition of ergodicity is not met though the Boltzmann and Gibbs entropies, they can be calculated, but their practical utility remains doubtful, and hence their equivalence with the thermodynamic entropy is not possible. However, in practice, we measure thermodynamic parameters to a fair degree of confidence in such systems. It means that the system executes a time averaging within the existence time of the nonequilibrium state. During this period, the members of the Ω or *W* which get sampled out cannot be identified beforehand. Hence, how to perform the commensurate ensemble averaging is not clear.
5. Thus, except for equilibrium states, nonequilibrium steady states, and the time-dependent non-equilibriun states with exceedingly small magnitudes of $\frac{dS}{dt}$, the Jaynes maximum entropy estimates should not be executed on Boltzmann and Gibbs entropies because of their uncertain practical utilities. And even if it is executed, its practical utility will need to be weighed properly. A similar situation might exist in the other scientific fields wherein the Jaynes maximum entropy estimate is employed; hence, caution needs to be exercised.

Indeed, it will be of interest to carry out corresponding computational investigations to clearly demonstrate what the physicality of the significantly large magnitude of $\frac{dS}{dt}$ is, which will demarcate the region of nonequivalence between statistical and thermodynamic entropies. It is also worth exploring whether the alternative Gibbs entropy of Equation (14) can be coincided with the thermodynamic entropy for very short lived nonequilibrium states. But that is a project in itself, and we plan to direct our future efforts along these lines.

## Data Availability

No data is involved in this presentation.

## Abbreviations

The following abbreviation is used in this manuscript:

CSTR	Continuously stirred tank reactor
